# The exceptional genomic word symmetry along DNA sequences

**DOI:** 10.1186/s12859-016-0905-0

**Published:** 2016-02-03

**Authors:** Vera Afreixo, João M. O. S. Rodrigues, Carlos A. C. Bastos, Raquel M. Silva

**Affiliations:** Department of Mathematics, University of Aveiro, Campus Universitário de Santiago, Aveiro, Portugal; Department of Electronics, Telecommunications and Informatics, University of Aveiro, Campus Universitário de Santiago, Aveiro, Portugal; Department of Medical Sciences and Institute of Biomedicine – iBiMED, University of Aveiro, 3810-193 Aveiro, Portugal, Campus Universitário de Santiago, Aveiro, Portugal; IEETA-Institute of Electronic Engineering and Informatics of Aveiro, Campus Universitário de Santiago, Aveiro, Portugal

**Keywords:** Exceptional symmetry, Genome, Chargaff’s second parity rule, Window analysis

## Abstract

**Background:**

The second Chargaff’s parity rule and its extensions are recognized as universal phenomena in DNA sequences. However, parity of the frequencies of reverse complementary oligonucleotides could be a mere consequence of the single nucleotide parity rule, if nucleotide independence is assumed. Exceptional symmetry (symmetry beyond that expected under an independent nucleotide assumption) was proposed previously as a meaningful measure of the extension of the second parity rule to oligonucleotides. The global exceptional symmetry was detected in long and short genomes.

**Results:**

To explore the exceptional genomic word symmetry along the genome sequences, we propose a sliding window method to extract the values of exceptional symmetry (for all words or by word groups). We compare the exceptional symmetry effect size distribution in all human chromosomes against control scenarios (positive and negative controls), testing the differences and performing a residual analysis. We explore local exceptional symmetry in equivalent composition word groups, and find that the behaviour of the local exceptional symmetry depends on the word group.

**Conclusions:**

We conclude that the exceptional symmetry is a local phenomenon in genome sequences, with distinct characteristics along the sequence of each chromosome. The local exceptional symmetry along the genomic sequences shows outlying segments, and those segments have high biological annotation density.

## Background

Chargaff’s first parity rule states that, in any sequence of double-stranded DNA molecules, the total number of complementary nucleotides is exactly equal [1]. Chargaff’s second parity rule states that those quantities are almost equal in a single strand of DNA [2–4], and this phenomenon holds in almost all living organisms.

The extension to the second parity rule is also known as single strand symmetry phenomenon. The single strand symmetry states that, in each DNA strand, the proportion of an oligonucleotide should be similar to that of its reverse complement [5–8]. There is no knowledge about why the parity is needed and there is no consensual explanation for the occurrence of the single strand phenomenon. There are some attempts to explain the phenomenon related with the species evolution process, for example: stem-loops hypothesis [9]; duplication followed by inversion hypothesis [10]; inversions and inverted transposition hypothesis [11]; no strand bias [12]; original trait of the primordial genome [8].

Powdel and others [13] studied the symmetry phenomenon in non-overlapping regions of DNA of specific size. They analysed the frequency distributions of the local abundance of oligonucleotides along a single strand of DNA, and found that the frequency distributions of reverse complementary oligonucleotides tend to be statistically similar. Afreixo et al. [14] introduced a new symmetry measure, which emphasizes that the frequency of an oligonucleotide is more similar to the frequency of its reverse complement than to the frequencies of other equivalent composition oligonucleotides. They also identified several word groups with a strong exceptional symmetry. Here, we have applied this measure to find genomic regions with very strong exceptional symmetry effect and to characterize their non-uniform behaviour. We observed exceptional symmetry throughout the human genome. Moreover, some regions showed outlying exceptional symmetry, and those are enriched in protein-coding annotated genes.

## Methods

### Materials

We analysed the whole human genome, reference assembly build 37.3, available from the website of the National Center for Biotechnology Information. In our data processing, the chromosomes were processed as separate sequences, words were counted with overlap. We also produced and used random control experiments. Those experiments tried to mimic some features of each human chromosome and contained the same number of base pairs of the corresponding chromosome (see ‘Control experiments’ subsection).

We obtained the coding sequences (cds file) for all the transcripts of the human genome (release 75) from Ensembl (http://www.ensembl.org/), to use in coding vs non-coding region classification.

### Exceptional genomic word symmetry

In a previous work, we proposed the concept of exceptional genomic word symmetry in equivalent composition groups (ECG), and globally [14]. Exceptional symmetry is a refinement of Chargaff’s second parity rule that highlights the words whose frequencies of occurrence are similar to those of their reversed complements, but are dissimilar to the frequencies of occurrence of other words with *equivalent composition*. Words of equal length are defined to have equivalent composition if they contain the same number of nucleotides A or T.

Some words are equal to their reverse complement. We denote these as self symmetric words (SSW). We also define a symmetric word pair as the set composed by one word *w* and the corresponding reverse complement word *w*^′^, with (*w*^′^)^′^=*w*.

Let *G*_*m*_ denote a set of words with equivalent composition, i.e. words containing the same number (*m*) of *A*s + *T*s. For words of length *k*=2, the ECGs are: *G*_0_={*CC*,*CG*,*GC*,*GG*}; *G*_1_={*AC*,*AG*,*CA*,*GA*,*CT*,*GT*,*TC*,*TG*} and *G*_2_={*AA*,*AT*,*TA*,*TT*}. The proposed exceptional symmetry measure for *G*_*m*_ is given by

(1) $$ \mathit{VR}(G_{m})= \sqrt{\frac{{X^{2}_{u}}(G_{m})/df_{u}(G_{m})+\epsilon}{{X^{2}_{s}}(G_{m})/df_{s}(G_{m}) +\epsilon}}, \;\;df_{s}>0   $$

where ${X^{2}_{s}}(G_{m})$ is used to evaluate the discrepancy between the frequencies of symmetric words in *G*_*m*_, and ${X^{2}_{u}}(G_{m})$ to evaluate the variability within *G*_*m*_ words (discrepancy from uniformity). To define those measures we establish the following notation

- *N*_*m*_ the number of elements in *G*_*m*_.
- $N^{\mathit {SSW}}_{m}$ the number of elements in *G*_*m*_ which are self symmetric words.
- ${N^{0}_{m}}$ the number of symmetric word pairs in *G*_*m*_, excluding the SSWs, such that both words in the pair are absent from the nucleotide sequence under study.
- *n*_*w*_ the frequency of occurrence of word *w* in a nucleotide sequence.
- *N*_*m*_ the frequency of occurrence of words from group *G*_*m*_ in a nucleotide sequence.

The discrepancy measures for equivalent composition group *G*_*m*_ can be described by

$$X^{2}_{s}(G_{m})=\left\{\begin{array}{ll}\frac{1}{2}\sum_{w \in G_{m} \wedge n_{w}+n_{w'}\not= 0}{\frac{(n_{w}-n_{w'})^{2}}{n_{w}+n_{w'}}} &n_{m}\not=0\\ 0,& n_{m}=0,\end{array}\right.$$

$$X^{2}_{u}(G_{m})=\left\{\begin{array}{ll} -n_{m}+N_{m}\sum_{w\in G_{m}}{\frac{{n_{w}^{2}}}{n_{m}}}, & n_{m}\not=0\\ 0,& n_{m}=0.\end{array}\right.$$

Taking into account that an SSW has no discrepancy from symmetry, we introduce here an adjustment to the degrees of freedom proposed in [14],

$$df_{u}(G_{m})=\left\{\begin{array}{ll} N_{m}-2,& n_{m}>0 \\ -1,& n_{m}=0. \end{array}\right.$$

and

$$df_{s}(G_{m})=\left(N_{m}-N^{\mathit{SSW}}_{m}-2{N^{0}_{m}}\right)/2-1 $$

According to the exceptional symmetry concept, if *VR*(*G*_*m*_)≈1, there is no exceptional symmetry, but if *VR*(*G*_*m*_)≫1, there is exceptional symmetry.

To measure the global exceptional symmetry, we use

(2) $$ \mathit{VR}= \sqrt{\frac{{X^{2}_{u}}/df_{u}+\epsilon}{{X^{2}_{s}}/df_{s}+\epsilon}}, \;\;df_{s}>0   $$

where ${X^{2}_{i}}=\sum _{m \in \{0,\ldots,k\}}{{X^{2}_{i}}(G_{m})}$, $df_{i}=-1+\sum _{m \in \{0,\ldots,k\}}{\left (df_{i}(G_{m})+1\right)}$ and *i*∈{*s*,*u*}.

The exceptional genomic word symmetry values were determined in all non-overlapping sub-chromosomal regions (windows) of several specific sizes (1000 bp, 2000 bp, 5000 bp and corresponding multiples of 10, up to the size of the chromosomes). The starting window size (1000 bp) was established taking into account the maximum word size under study (k = 10) and the expected number of words in each ECG assuming uniform word distribution: as expected value we fixed at least one word in the smallest ECGs, *G*_0_ and *G*_*k*_. However, note that for large *k*, the shorter windows (1000 bp and 2000 bp) may not include enough ocurrences in the smallest ECGs to provide a good estimate of *VR*(*G*_*m*_).

### Control experiments

To produce a negative control (without exceptional symmetry) we generated two types of random scenarios

- random (rnd): assuming independence and using the human chromosome nucleotide composition as input. There are small differences between the frequencies of occurrence of complementary nucleotides. Moreover, in this scenario the expected probabilities of the reverse complements are not equal but there are words in an ECG (e.g. *ATT*, *TAT*, *TTA*) with equal expected probabilities.
  **Input:** nucleotide probabilities (*π*_*A*_, *π*_*C*_, *π*_*G*_, *π*_*T*_, where *π*_*w*_ denotes the probability of *w*).
- random symmetric (sym): assuming independence and using the same composition for complementary nucleotides as input. In this scenario the expected probabilities of ECG words are the same.
  **Input:** nucleotide probabilities (*π*_*A*_, *π*_*C*_, *π*_*G*_, *π*_*T*_, subject to ${\pi _{w}}={\pi _{w^{\prime }}}\phantom {\dot {i}\!}$ with *w*∈{*A*,*C*,*G*,*T*}).

To produce a positive control (with exceptional symmetry for *k*=2) we generated two types of random scenarios

- random with first-order dependence (mrnd): assuming first order Markov structure using the human chromosome nucleotide and dinucleotide composition as inputs.
  **Input:** matrix of nucleotide transition probabilities ($\mathbf {P}= \,[\pi _{K_{1}K_{2}\phantom {\dot {i}\!}}/\pi _{K_{1}}]\phantom {\dot {i}\!}$ with *K*_1_, *K*_2_∈{*A*,*C*,*G*,*T*}) and initial probabilities (*π*_*A*_, *π*_*C*_, *π*_*G*_, *π*_*T*_).
- random exceptional symmetric with first-order dependence (msym): assuming first order Markov structure using the human chromosome nucleotide and dinucleotide composition and using the same composition for inverted complement dinucleotides as inputs.
  **Input:** matrix of nucleotide transition probabilities ($\mathbf {P}=\,[\pi _{K_{1}K_{2}}/\pi _{K_{1}}]$ with *K*_1_, *K*_2_∈{*A*,*C*,*G*,*T*}, subject to ${\pi _{w}}={\pi _{w^{\prime }}}\phantom {\dot {i}\!}$ with *w*∈{*AA*,*AC*,…,*TT*}) and initial probabilities (*π*_*A*_, *π*_*C*_, *π*_*G*_, *π*_*T*_, subject to ${\pi _{w}}={\pi _{w^{\prime }}\phantom {\dot {i}\!}}$ with *w*∈{*A*,*C*,*G*,*T*}).

### Coding region classification

We extracted the start and end positions of all known coding sequences from the Ensembl cds file whose gene biotype was “protein coding”. For genes with multiple transcripts, the gene start position was considered as the minimum start position of all the transcripts of that gene, and the end position as the maximum end position of the same transcripts. For each chromosome, for a given word length *k* and window size, windows that intercept a gene were labeled as coding neighbourhood windows, windows that do not intercept any gene were labeled as non-coding windows.

### Isochores region classification

We used the IsoSegmenter program [15] with the default parameters, to classify the human genome in isochore families: L1, L2, H1, H2, H3. For each chromosome, for a given word length *k* and window size, windows fully included in an isochore were labeled with the corresponding isochore family. Windows spanning more than one isochore were discarded.

### DNA segmentation procedure

In order to evaluate the association between the local exceptional symmetry values and their biological relevance we propose a threshold based method to perform DNA segmentation into high and low exceptional symmetry regions.

To perform the DNA segmentation on the exceptional symmetry profile (the sequence of exceptional symmetry values, also referred to as *VR* sequences), we need to choose an adequate window size and word length. The window size and the word length which show the widest diversity of local behaviours along the sequence have the potential to perform a good sequence segmentation. So, to explore the variability of local behaviours we evaluate the strict stationarity using the Kolmogorov Smirnov (KS) statistic.

To explore the stationarity, and find the window size and the word length which show the highest lack of stationarity, we propose the following procedure:

- the *VR* chromosome sequence (the sequence of *VR* values in each chromosome) is divided in successive non-overlapping subsequences (*VR* subsequences) with a fixed length (50, 100, 200);
- for each word length and for each window size, we compute the KS statistic between the *VR* distribution of each subsequence and the *VR* distribution of its complete chromosome sequence;
- to characterise the lack of stationarity in each exceptional symmetry experiment (defined by the window size and the word length) we compute the average of all KS statistics obtained from *VR* subsequences.
- the window size and word length of the exceptional symmetry experiment with the highest average of KS values are chosen.

To perform the DNA segmentation

- we determined the quartiles of the *VR* chromosome sequence;
- we calculated the outlier threshold as the third quartile (*Q*_3_) plus 1.5 times the interquartile range (*IQR*): *Q*_3_+1.5∗*IQR*;
- we identified the windows with *VR*≥*Q*_3_+1.5∗*IQR* as the regions with very high local exceptional symmetry (outlying regions) and the other regions with *VR*<*Q*_3_+1.5∗*IQR* as the regions without very high local exceptional symmetry (non-outlying regions).

### Functional annotation enrichments

Using BioMart, we extracted the annotation information for Homo sapiens genes (GRCh37.p13) dataset from the Ensembl Genes database. To examine the functional annotation enrichments of outlying regions vs non-outlying regions we computed the annotation density ratio (*ADR*) for each chromosome, defined by

(3) $$ \mathit{ADR}=\frac{\frac{n^{A}_{\text{outlying segments}}}{\sum{\text{outlying segments length}}}}{\frac{n^{A}_{\text{non-outlying segments}}}{\sum{\text{non outlying segments length}}}}   $$

where ${n^{A}_{S}}$ denotes the number of annotations in the subset *S*. We used the chi-square test to evaluate if the annotations are equally distributed in the two subsets. To better evaluate the diferences between both subsets, we used the adjusted residual analysis. Under the homogeneity hypothesis the adjusted residuals have a standard normal distribution [16].

## Results and discussion

In this study, we analysed local exceptional word symmetry in the complete human genome. In particular, we analysed words of lengths up to 10 in all human chromosomes. We performed a sliding window analysis in terms of exceptional symmetry (*VR*). We obtained, when possible, results for the following window sizes: 10^*l*^, 2×10^*l*^, 5×10^*l*^ base pairs, with *l*∈{3,4,5,6,7,8}.

We performed our analysis using five ACGT sequence types: real human chromosomes, and corresponding simulated sequences generated according to four distinct random scenarios. For each fixed window size and word length we determined the exceptional symmetry (*VR*) and symmetry $\left ({X^{2}_{s}}\right)$ values. Each of these experiments is characterized by median and median absolute deviation values.

To evaluate the effect of chromosome type, window size and word length on the local exceptional symmetry behaviour, we considered the window *VR* median values of each ACGT sequence (chromosomes or corresponding random chromosomes).

**Fig. 1:**
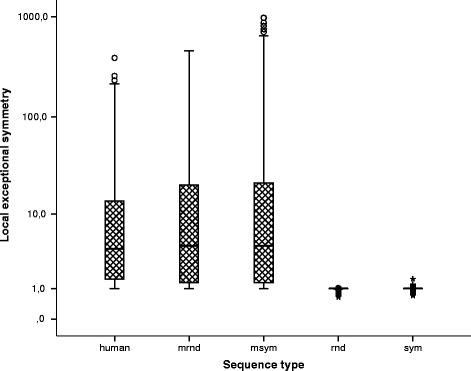
Local exceptional symmetry by sequence type in the human genome. Box plots comparing the local exceptional symmetry values (*V* *R* median) using all chromosomes, window length, and word length results, separated by: human chromosomes (human), random scenarios with first order structure (without exact symmetry: mrnd, and with exact symmetry for k = 2: msym), and random scenarios assuming nucleotide independence (without exact symmetry: rnd, and with exact symmetry for k = 1: sym). The local exceptional symmetry in the human genome and positive control experiments (mrnd and msym) is higher than in the random scenarios produced without exceptional symmetry (rnd and sym)

Figure 1 shows five boxplots; one for each sequence type. The local exceptional symmetry in the human genome is clearly higher than in the random scenarios produced without exceptional symmetry (rnd and sym), but globally the effect is similar to random sequences generated with first order Markov models (mrnd and msym).

**Fig. 2:**
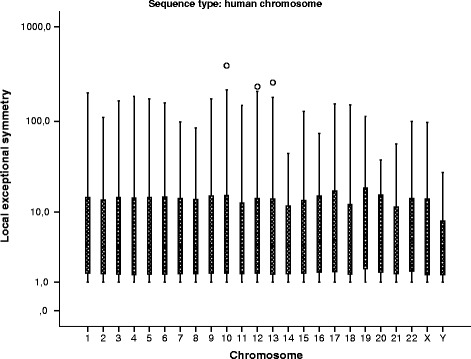
Local exceptional symmetry by human chromosome. Box plots comparing the local exceptional symmetry values (*V* *R* median) by human chromosomes for all window and word lengths. The results are similar across all chromosomes

Local exceptional symmetry has no significant differences between chromosomes (Kruskal-Wallis test *p*≫0.1). Figure 2 presents the results of the local exceptional symmetry using boxplots for comparing the various human chromosomes. The similarity of the chromosomes results is easily observed in the plot.

**Fig. 3:**
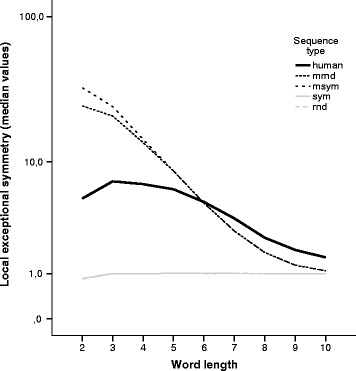
Local exceptional symmetry by word length. Line plot comparing the median of local exceptional symmetry values (*V* *R* median) by word length, for human and random scenarios. The sym and rnd scenarios overlap. The local exceptional symmetry in the human genome decreases with increasing word length, excluding *k*=2. The effect of word length in local exceptional symmetry is significantly different in human and random scenarios. For *k*≥7 the local exceptional symmetry in the human genome surpasses that obtained by positive control scenarios

Figure 3 plots the median of the local exceptional symmetry values by word length using all chromosomes and all window size data. Excluding *k*=2, the local exceptional symmetry and the corresponding dispersion decrease with increasing word length. The effect of word length in local exceptional symmetry has significantly different behaviours in human and random scenarios (random and symmetric random). As was expected, for shorter word lengths we obtain higher local exceptional symmetry values in random scenarios with first order dependence structure, but for *k*≥7 the human chromosomes surpass the random values. In Fig. 3 all chromosomes results are combined, but the local exceptional behaviour is also present in each chromosome.

### Window size effect

**Fig. 4:**
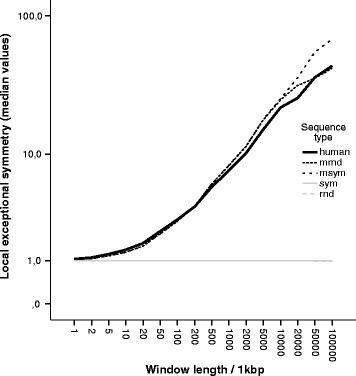
Local exceptional symmetry by window length. Line plot comparing the median of local exceptional symmetry values (*V* *R* median) by window length, for human and random scenarios. The local exceptional symmetry increases with increasing window length, except for the negative control scenarios (sym and rnd)

Figure 4 shows the local exceptional symmetry values by window size. In the presence of exceptional symmetry, the local exceptional symmetry values increase with the window size, as was expected.

The random sequences msym and mrand were generated with forced exceptional symmetry under stationary behaviour, and an increasing tendency was observed on their local exceptional symmetry values as a function of the window size. For the random sequences without exceptional symmetry (sym and rnd) the local *VR* values are nearly constant (see Fig. 4).

All human chromosomes exhibit increased exceptional symmetry with increasing window lengths. In general, the behaviour is similar to the random sequences with first order Markov structure. However, we can observe higher values in the first order Markov sequences than in the human sequences.

### Local exceptional symmetry stationarity

**Fig. 5:**
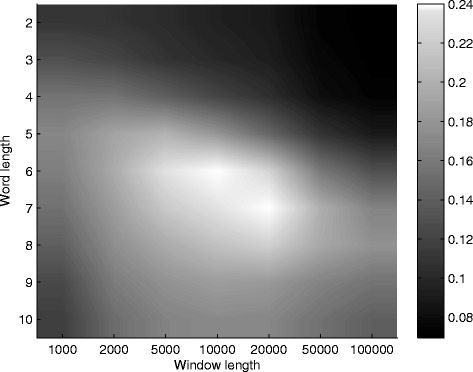
Heat map for Kolmogorov-Smirnov statistics. Kolmogorov-Smirnov statistics by word length and window size, using *V* *R* subsequences of length 200, for the complete human genome. The human genome shows non-stationary local exceptional symmetry behaviour, with maximum value for *k*=7 and window length equal to 20,000 bp

In order to find the window size and the word length with the highest potential to show distinct local behaviour along the sequence, we explored the stationarity using the procedure described previously. Figure 5 presents a heat map of the results of the Kolmogorov-Smirnov statistic by word length and by window size in the human genome, obtained with *VR* subsequences with length 200. The results obtained with *VR* subsequences with length 50 and 100 are similar to these (not shown). The human genome shows non stationary local exceptional symmetry behaviour. Local results are distinct from the global. We observe the maximum value for *k*=7 and for window size equal to 20,000 base pairs. The second highest value is obtained for *k*=6 and window size equal to 10,000 base pairs.

### Local exceptional ECG symmetry

As *G*_0_ and *G*_*k*_ are the sets with fewer elements, higher variability in *VR* results is expected, and this was confirmed in all sequences under study (results not shown). We verified that almost all human ECGs have higher *VR* values than the random scenarios.

**Fig. 6:**
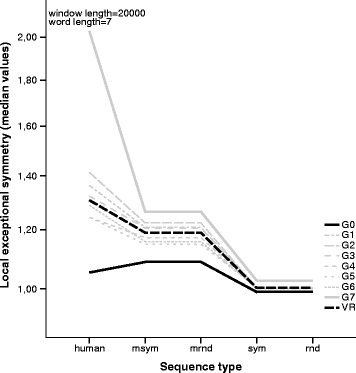
Local exceptional symmetry by ECG. Line plot comparing local exceptional symmetry median values by ECG for *k*=7 and 20,000 bp window length in the human genome and random scenarios. ECG *G*_7_ has the highest local exceptional symmetry values and *G*_0_ has the lowest

Figure 6 shows the comparison of the human and random local ECG exceptional symmetry results for word length 7 and window length 20,000. In the human genome, the ECG *G*_7_ has the highest local exceptional symmetry values (and dispersion). Surprisingly, the human *G*_0_ has lower median *VR* values than the random sequences that incorporate exceptional symmetry.

### Segmentation

**Fig. 7:**
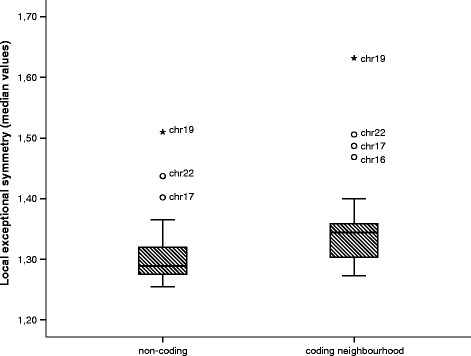
Chromosome median exceptional symmetry values. Box plots comparing the local exceptional symmetry median values by chromosome in coding neighbourhood windows and in non-coding windows. The analysis was performed with *k*=7 and 20,000 bp window length. Chromosome 19 has the highest median *V* *R* value in both window sets. The coding neighbourhood windows show significantly higher *V* *R* values than non-coding windows

**Fig. 8:**
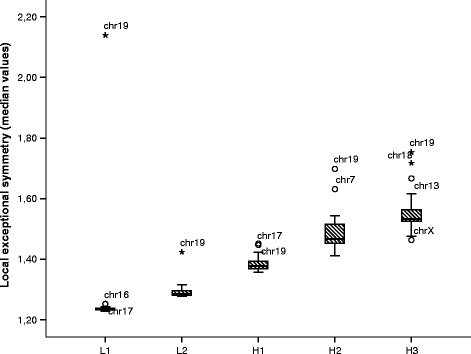
Chromosome median exceptional symmetry values. Box plots comparing the local exceptional symmetry median values by chromosome in five isochore families: L1, L2, H1, H2 and H3. The analysis was performed with *k*=7 and 20,000 bp window length. Chromosome 19 has outlying median *V* *R* values in all isochore families. The H isochores show significantly higher *V* *R* values than L isochores

We have observed exceptional symmetry throughout the genome, including coding and non-coding regions. Figure 7 shows the chromosome median exceptional symmetry values for *k*=7 and window length 20,000, divided in two sets: the coding neighbourhood windows (70,646 *VR* subsequences), and the non-coding windows (72,543 *VR* subsequences). The coding neighbourhood windows show significantly higher *VR* values than non-coding windows (*p*<0.001, z-test). However, the effect size of the difference is small (Cohen’s *d*≈0.2). Figure 8 presents box plots comparing the local exceptional symmetry median values for *k*=7 and 20,000 bp window length in the five isochore families: L1, L2, H1, H2, H3. The exceptional symmetry effect between H and L isochores show strong and significant diferences (*p*<0.001, *d*>0.8).

**Fig. 9:**
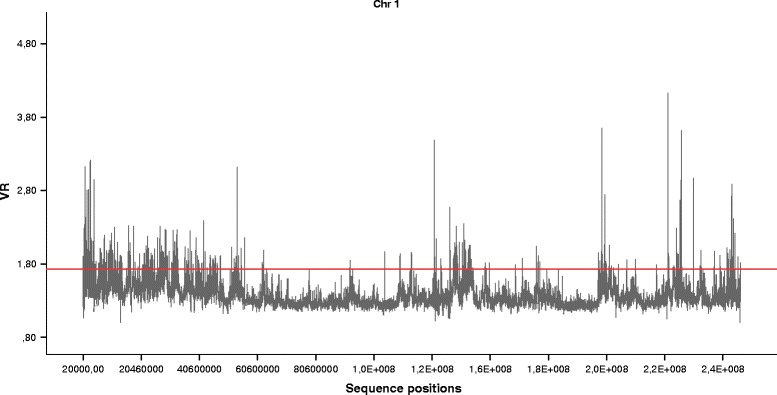
Chromosome 1 segmentation. Chromosome 1 *V* *R* results for *k*=7 and 20,000 bp window size. The horizontal line shows the threshold for segmentation into very high local exceptional symmetry regions (outliers) and complementary regions (non-outliers)

Additionally, there are several windows with strong outlying behaviour. We applied the outlier detection procedure described previously. As an example, Fig. 9 shows the local symmetry results for chromosome 1. Table 1 shows the percentage of outlying segments by chromosome. In all chromosomes, the percentage of outliers is less than 10 %.

**Table 1:** Outlying segments description by human chromosome for *k*=7 and 20,000 window size

Chr	1	2	3	4	5	6	7	8	9	10	11	12
Outlying segments %	4.8	6.1	6.4	7.6	7.7	6.5	9.1	6.8	4.7	5.3	6.0	6.5
*A* *D* *R*	3.5	3.9	4.1	4.3	3.8	3.6	2.9	3.5	4.0	3.3	3.3	6.9
*χ* ^2^ test (p-value)	**	**	**	**	**	**	**	**	**	**	**	**
*ϕ*∗100 %	9	9	10	14	13	14	12	14	13	12	13	12
Chr	13	14	15	16	17	18	19	20	21	22	X	Y
Outlying segments %	5.6	4.5	4.4	3.5	3.0	6.7	1.5	5.5	5.0	2.6	8.6	7.0
*A* *D* *R*	5.5	4.3	3.8	3.1	2.2	4.5	1.2	2.5	5.2	2.5	3.8	1.5
*χ* ^2^ test (p-value)	**	**	**	**	**	**	**	0.003	**	1	**	0.038
*ϕ*∗100	16	15	11	7	6	14	8	9	15	8	14	19

The p-values are adjusted for Holm–Bonferroni method

^**^means that the p-value is lower than 0.001

*A* *D* *R* - annotation density ratio; *χ* ^2^ test - p-value of chi-square test; *ϕ* - phi measure

### Annotation results

To characterize the chromosome features associated with the outlying segments of local exceptional symmetry, we have performed annotation enrichment analyses. Table 1 presents the annotation density ratio (*ADR*, Eq. 3) of the outlying segments vs non-outlying segments by chromosome, as defined by the DNA segmentation procedure described in the ‘Methods’ section. We observe that in the human genome the *ADR* values are higher than 1 for all chromosomes, which means that the density of annotation in outlying segments is higher than in non-outlying segments (average value equal to 3.6 and standard deviation equal to 1.2). Table 1 also shows the p-values of the chi-square test for homogeneity of annotation types between outlying and non-outlying segments. The p-values were adjusted using the Holm–Bonferroni method [17]. Almost all chromosomes display significant diferences in annotation between segment types (outlying vs non-outlying). In chromosome 22, however, the difference was not considered significant, perhaps due to the low percentage of outlying segments and chromosome size. Still, the dissimilarity effect between outlying and non-outlying annotations is present in all chromosomes (phi measure (*ϕ*) range between 0.06 to 0.19).

**Fig. 10:**
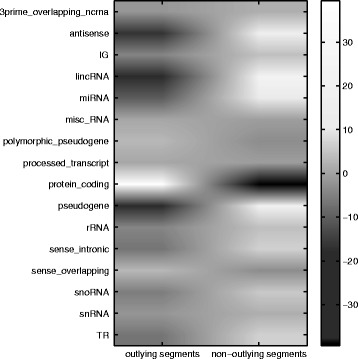
Association residual analysis between gene type annotation and segment type. Heat map of adjusted residuals by each gene and segment type for all human chromosomes data. The counts of protein-coding gene annotations in outlying segments are significantly larger than expected, whereas in non-outlying segments long intergenic non-coding RNAs (lincRNA), microRNAs (miRNAs), antisense and pseudogene annotations predominate

Figure 10 presents a heat map with the adjusted residuals of the homogeneity in gene type annotation using all chromosome sequences. The counts of protein-coding gene annotations in outlying segments are significantly larger than expected (adjusted residual equal to 37.7), whereas in non-outlying segments long intergenic non-coding RNAs (lincRNA), microRNAs (miRNAs), antisense and pseudogene annotations predominate (adjusted residuals equal to 22.0, 9.6, 15.5 and 20.3, respectively).

## Conclusion

The local exceptional symmetry profile provides a numerical signature along genomic sequences. The proposed procedure to analyse local exceptional symmetry in the human genome can be applied to any genomic sequence as a segmentation procedure and also as a genomic signature. The results obtained in this work suggest that for the human genome there is an optimal word length and window size to explore the local exceptional symmetry (7 and 20,000 bp, respectively).

The local exceptional symmetry in the human genome is very dissimilar from random scenarios (both with independent symbols or first order Markov structure) showing, as expected, a non-stationary behaviour. Globally, the human genome exhibits high local exceptional symmetry values, which for some word lengths are lower than the values for positive control experiments, but higher than the values for negative control experiments.

The global statistical pattern (location and dispersion values), which is obtained from the exceptional symmetry profiles, is present in all chromosomes of the human genome. The local profile is chromosome specific and the regions with very high exceptional symmetry values are strongly associated with the presence of protein coding genes, although non-coding regions also present exceptional symmetry. Additionally, the local exceptional symmetry values are positively correlated with the GC content as defined by isochore families.
